# Time-Integrated Position Error Accounts for Sensorimotor Behavior in Time-Constrained Tasks

**DOI:** 10.1371/journal.pone.0033724

**Published:** 2012-03-21

**Authors:** Julian J. Tramper, Bart van den Broek, Wim Wiegerinck, Hilbert J. Kappen, Stan Gielen

**Affiliations:** Department of Biophysics, Donders Institute for Brain, Cognition and Behaviour, Radboud University Nijmegen, Nijmegen, The Netherlands; Bielefeld University, Germany

## Abstract

Several studies have shown that human motor behavior can be successfully described using optimal control theory, which describes behavior by optimizing the trade-off between the subject's effort and performance. This approach predicts that subjects reach the goal exactly at the final time. However, another strategy might be that subjects try to reach the target position well before the final time to avoid the risk of missing the target. To test this, we have investigated whether minimizing the control effort and maximizing the performance is sufficient to describe human motor behavior in time-constrained motor tasks. In addition to the standard model, we postulate a new model which includes an additional cost criterion which penalizes deviations between the position of the effector and the target throughout the trial, forcing arrival on target before the final time. To investigate which model gives the best fit to the data and to see whether that model is generic, we tested both models in two different tasks where subjects used a joystick to steer a ball on a screen to hit a target (first task) or one of two targets (second task) before a final time. Noise of different amplitudes was superimposed on the ball position to investigate the ability of the models to predict motor behavior for different levels of uncertainty. The results show that a cost function representing only a trade-off between effort and accuracy at the end time is insufficient to describe the observed behavior. The new model correctly predicts that subjects steer the ball to the target position well before the final time is reached, which is in agreement with the observed behavior. This result is consistent for all noise amplitudes and for both tasks.

## Introduction

In the past decade, it has become clear that many properties of human motor coordination can be well explained using the framework of stochastic optimal feedback control [1]–[4]. Successful applications have been reported for the manipulation of objects [1], [5], the stability to accuracy trade-off [6], bimanual responses to perturbations [7], visual feedback during hand movements [8], [9], cooperation between players [10], risk sensitivity [11], [12], and adaptation [13]–[15]. This framework uses optimization techniques to find a control law that minimizes a cost function associated with the actions necessary to perform a specific task. This cost function is closely related to what the system is trying to achieve. For sensorimotor control, the cost function therefore includes a component which is related to the effort necessary to complete the goal. The value of this effort-related penalty component, the control cost, increases quadratically with the magnitude of the control signal. In addition, the cost function generally includes a component related to the performance of the task. For goal directed movements, task performance can be modeled by including an end cost which penalizes the squared difference between the position of the effector (e.g., hand or cursor on a screen) and the goal at the end of the trial [16]. This end cost component thus reflects the accuracy in achieving the goal.

In daily life we have to make decisions based on limited information from a noisy environment. Not only must we decide *what* the optimal sequence of actions should be, we must also decide *when* these actions should be executed. This is especially true for motor tasks in which subjects are required to complete the task within a particular time interval. For example, a tennis player has to hit the ball at the right angle and within a narrow time interval where the ball is within the reach of the player. Several studies have investigated such time-constrained motor tasks [5]–[7], [9], [11]–[13]. Typically, the observed behavior has been modeled by minimizing a weighted combination of the control cost and the end cost, which corresponds to minimizing the effort and maximizing the task performance. Such a model predicts that subjects arrive on target exactly at the final time. Another strategy might be that subjects try to reach the target position well before the final time to avoid the risk of missing the target. To test this, we have investigated whether minimizing the effort and maximizing the task performance is sufficient to predict human motor behavior in time-constrained sensorimotor tasks. In addition to this standard model, we postulate a new model that includes an additional cost criterion which penalizes deviations between the position of the effector and the target throughout the trial, forcing arrival on target well before the final time.

To investigate which model gives the best fit to the data and whether that model is generic, we tested the models in two different time-constrained tasks. In the first task, subjects had to control a joystick to move a ball on a screen to hit a target at the end of a fixed time interval. In the second task, the single target was replaced by two targets. Now subjects were asked to steer the ball to one of the targets. They were free to choose which one. Previous work has shown that the subject's behavior may depend on the level of uncertainty in the task [11], [12]. Therefore, we superimposed noise with different amplitudes on ball position to introduce uncertainty about future ball positions. This allowed us to investigate the ability of the models to predict motor behavior in two different tasks and at different levels of uncertainty.

The results show that a simple cost function representing a trade-off between effort and performance is insufficient to describe the observed behavior in our experiment. The model that we propose predicts that subjects steer the ball to the target position well before the final time is reached, which is in agreement with the observed behavior. This result is consistent for all noise amplitudes and for both tasks.

## Methods

### Subjects

We have tested subjects in two different tasks. Twelve subjects (seven males) aged between 21 and 28 years participated in these tasks. Four of them (labeled as S1–S4) took part in the one-target task, four (S9–S12) in the two-target task and four (S5–S8) in both tasks. All subjects were right-handed and none of them had any known neurological or motor disorder. All subjects were naive regarding the purpose of the experiment. Subjects gave written informed consent prior to the start of the experiment according to institutional guidelines of the local ethics committee. We have submitted the protocol for our experiments to the “Commissie Mensgebonden Onderzoek” (or CMO, translated: “Committee for research on human subjects”) of the Radboud University Medical Center Nijmegen, which approved the experiments in our study.

### Experimental procedures

Subjects were seated in a chair, such that their eyes were located 70 cm in front of a 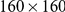 cm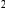 rear-projection screen. A white ball and one or two white targets on a dark background were rear projected on the screen with an LCD projector (JVC DLA-S10) with a refresh rate of 75 Hz. The ball and target were represented by a dot with a diameter of 1.5 cm and a vertically oriented bar with a size of 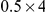 cm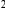, respectively. The ball moved from the left to the right at a constant velocity and subjects could control the vertical velocity of the ball by a joystick. The joystick could only move up- or downward in a range between 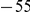 and 55 deg. The length of the joystick handle was 17 cm. Joystick output was measured at a sample rate of 75 Hz. To avoid a bias in the ball's movement when the joystick was near the neutral position, the output was set to zero for excursions in the range between 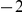 and 2 deg. Above (below) this threshold, the velocity signal increased (decreased) linearly with joystick excursion angle.

The size of the computer generated animated scene on the screen was 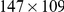 cm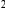. We defined a coordinate system with time 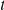 in the horizontal direction, where the left and right boundaries of the animation corresponded to the start (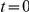) and end (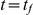) of each trial. The range 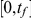 corresponded to a distance of 147 cm. The vertical direction was represented by normalized coordinates 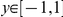, where 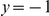 and 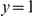 defined the lower and upper boundary of the animation, respectively. The maximum excursions of the joystick corresponded to a control 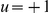 (upward) and 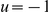 (downward).

**Figure 1:**
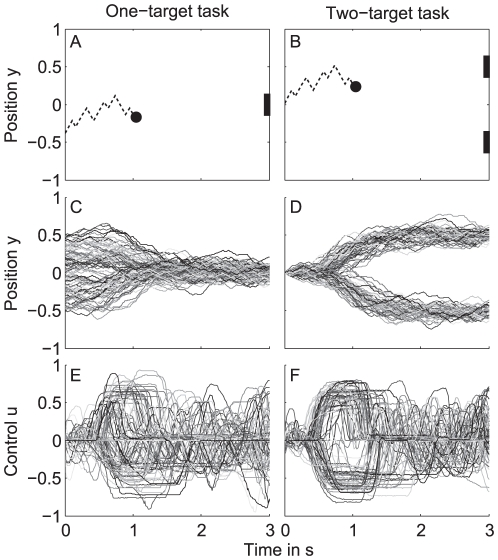
Schematic of the tasks. (a) In the one-target task (left panel), subjects had to control a joystick to move a ball on a screen to hit a target (rectangle) at time 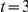 s. The ball (circle) started at a random vertical position between 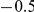 and 0.5 at the left of the screen and moved at a constant horizontal velocity to the right. Subjects could move the ball up- or downwards. Gaussian white noise was superimposed on the vertical ball position to introduce uncertainty about future ball positions. The dashed line illustrates the trajectory of the ball. (b) In the two-target task (right panel), two targets were present at vertical positions 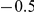 and 0.5. The ball started at vertical position 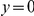 at the left of the screen. Subjects were asked to steer to one of the targets and they were free to choose which one. All other experimental conditions were exactly the same as for the one-target task. (c) Ball position time traces (100 trials) of subject S6 performing the one-target task with noise amplitude 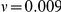. (d) Same for the two-target task. (e) Control signal time traces corresponding to the ball position time traces in panel c (100 trials) of subject S6 performing the one-target task with noise amplitude 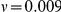. (f) Same for the two-target task.

In the one-target task, the target was located in the middle at the right of the screen (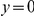, see Figure 1a). The ball started at the left side of the screen at a random vertical position between 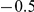 and 0.5 and moved at a constant horizontal velocity of 49 cm/s to the right, reaching the right boundary after three seconds (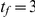 s). The subject's task was to hit the target by steering the ball up- or downwards. Thus, the subject was unable to manipulate the horizontal position of the ball. No instruction was given as to whether the movement should meet any criteria, except to hit the target with the ball.

Gaussian white noise was superimposed on the vertical position of the ball to introduce uncertainty about future positions. Thus, in the absence of a control signal the displacement of the ball described a Wiener or ‘random walk’ process. At each time step, the vertical position 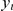 at time 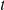 was updated according to 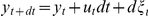, with 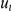 the subject's control signal at time 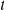 (units s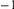), 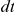 the time step size (13.3 ms), and 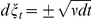 pseudo-random Gaussian white noise at time 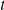 with amplitude 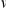 (units s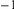). Subjects were tested in four consecutive blocks with 100 trials each, with a noise amplitude 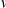 of consecutively 0 (no noise), 0.009, 0.04, and 0.08. For each noise amplitude, the same pseudo-random sequences were used such that each subject was subjected to exactly the same noise realizations. Each block with 100 trials was preceded by five trials to familiarize subjects with the task. These five trials were not included in the data analysis.

In the two-target task, we replaced the single target by two targets at vertical positions 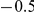 and 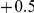 (see Figure 1b). In each trial, the ball started at vertical position 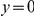. Subjects were now asked to steer the ball to one of the targets. They were free to choose which one. All other experimental conditions were exactly the same as for the one-target task.

### Standard model with end cost only

We used stochastic optimal feedback control to predict the subject's control and corresponding position of the ball for each noise amplitude and for both tasks. The dynamics of the control problem was described by the stochastic differential equation
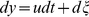
(1)with 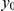 and 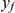 the initial and final ball position along the vertical axis. Equation 1 shows that a change in vertical position 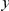 was caused by a control action 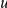 and noise 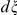. We defined a cost function with two components. First, we included the cumulative control cost proportional to the integral of the square of the control during the trial, which is defined by
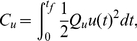
(2)where 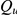 is a positive constant. Second, we added an end cost proportional to the squared difference between the ball's end position 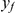 and the target position 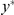, which is defined by
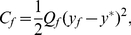
(3)where 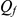 is a positive constant. The optimal control problem was to find the control sequence 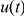 which minimizes the sum of the control cost 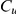 and the end cost 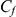. For the one-target task, the optimal control solution can be solved analytically [17]. Because the dynamics was stochastic (except for the case with noise amplitude 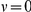), we consider the expectation value of the cost function over all possible future realizations of the Wiener process, which we minimize over all possible controls:
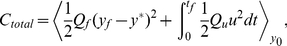
(4)where the first and second component represent the end cost and control cost, respectively. The subscript 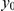 on the expectation value indicates an expectation over all stochastic trajectories starting in 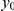. For this problem, the optimal cost-to-go 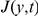 at time 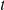 and position 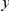 can be computed exactly [17], and is given by
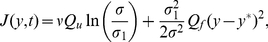
(5)with 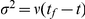 and 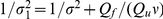. The optimal control is proportional to the partial derivative of 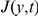 to 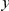 [17]:
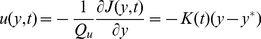
(6)with
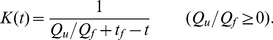
(7)where 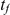 is the trial duration (final time). Equations 6 and 7 show that the optimal control 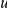 increases with increasing deviation from the final position 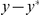, and with 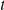 getting closer to 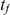. Note that these theoretical predictions imply that the optimal control is independent of the noise amplitude 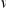.

To derive the optimal control solution for the two-target task we consider the same dynamical system as for the one-target task (equation 1). In the second task the system had to reach one of two targets at locations 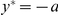 and 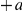 at a future time 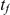. For this task, the end cost is defined by
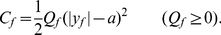
(8)


The optimal control is given by
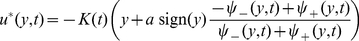
(9)with
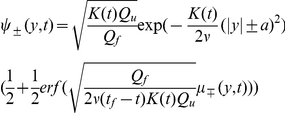
and
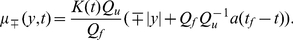
(10)


(see *Appendix S1* for the derivation). The optimal control in the noiseless condition (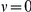) is obtained by taking the limit 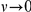 which gives
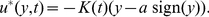
(11)


For 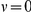, this model shows that the optimal strategy would be to steer the ball in a straight line from the initial position to the nearest target by exerting a constant control, in agreement with the prediction of deterministic optimal control [18].

### Extended model with position cost

The standard model described above assumes that the optimal control solution can be found by minimizing a cost function with a control cost and end cost component, which corresponds to a compromise between minimizing the subject's effort and maximizing the performance. In this study, we investigate whether such a model is sufficient to account for human motor control in a task where subjects have to reach a goal within a particular time interval 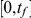. Therefore, we extended the standard model by introducing an additional cumulative cost that penalizes deviations between ball position and target position during the trial. This cost criterion tends to steer the ball to the vertical target position 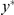 well before the final time 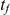 and is defined by
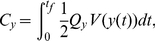
(12)where
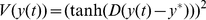
(13)for the one-target task where the target was located at 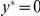, and
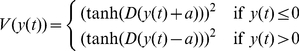
(14)for the two-target task where the two targets were located at 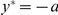 and 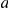. The parameters 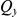 and 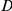 have positive values. 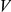 is zero when the ball's vertical position is equal to the target position. It increases approximately quadratically when the distance between ball and target is small and less than quadratically for larger distances. The position cost is constrained to a finite value when the distance between ball and target is large, eliminating the effect of outliers. This particular shape of 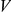 was chosen because previous experiments on sensorimotor learning showed that subjects implicitly use a cost function which is subquadratic for large errors [19], i.e., outliers tend to be ignored. This extended model minimizes the expected cost that is now given by
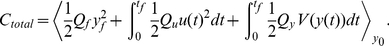
(15)


The optimal expected cost-to-go at time 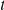 and position 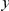 can be written as
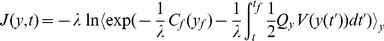
(16)where 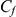 is the end cost function, 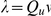, and 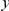 satisfies the uncontrolled dynamics 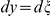 [17]. The optimal control was calculated by taking the gradient of the optimal expected cost-to-go. A closed form solution for the optimal expected cost-to-go in general does not exist. Therefore, we inferred the optimal control solution approximately by taking the following approach. From the dynamic programming principle [20] and equation 16 it follows that the optimal expected cost-to-go satisfies
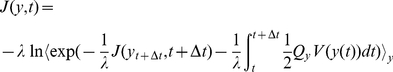
(17)for any time step 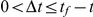. We approximated this equation by
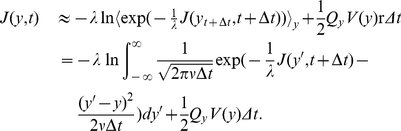
(18)


We set 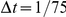 s since the sample rate in the experiments was 75 Hz. With equation 18, we computed the optimal expected cost-to-go at any time prior to the end time, starting at the end time where 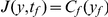, and then going backwards in time steps of size 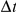. We approximated the spatial integral in equation 18 by a sum which was obtained by discretizing space into steps of size 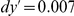. The optimal control was derived by taking the gradient of the optimal expected cost-to-go:
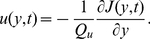
(19)


Since the cost function of this model contains the position cost as an additional component, we call this model the ‘extended model’. We call the model with end cost only the ‘standard model’. The extended model gives the same solution as the standard model if the position cost parameter 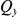 is zero (equations 12 and 13). For 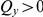, the extended model gives solutions which cannot be obtained by the standard model.

### Data analysis

In most of the trials, subject S5 showed a fragmentary, discontinuous control signal in which periods of high control were alternated with periods of no control throughout the trial. This was especially true for the noiseless condition. The other subjects showed a rather smooth, continuous control signal in most of the trials. Since the optimal control models assume a continuous control signal, subject S5 was excluded from the analysis.

For each task, subject, and noise amplitude we determined the model performance of the extended model with position cost and that of the standard model with end cost only using a 100-fold cross-validation. From each block with 100 trials, 50 trials were randomly drawn. From this subset of 50 trials, 45 trials were randomly selected to train both models to find the optimal model parameters. This was done by minimizing the mean square error (MSE) between the optimal control 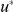 according to the model and the actual control 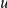 in the training data:
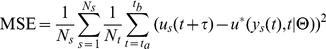
(20)where 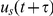 is the control according to the data at time 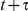 in trial 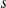, 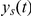 is the position of the ball at time 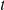 in trial 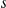, 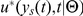 is the model control at time 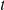 and position 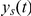 given the model parameters 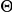, and 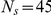 represents the number of trials. For both models we set 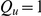, resulting in 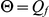 for the standard model and 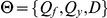 for the extended model. We introduced a sensorimotor delay 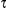 of 200 ms to take into account the time that it typically took for subjects to respond to changes in ball position during the task [21], [22]. The results did not depend crucially on the value of the time delay (see *Discussion*). The summation over time 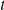 runs from 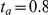 s to 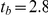 s, which corresponds to 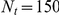 since the sample rate was 75 Hz. The lower bound of 0.8 s was chosen to include only responses well after the subjects' reaction time, which had a median value of 0.47 s (25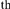 and 75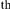 percentile was 0.31 s and 0.68 s, respectively). The upper bound of 2.8 s was chosen to account for the sensorimotor delay (0.2 s). Each model was tested on the remaining five trials, yielding a test error for the standard and the extended model. The test error was computed from equation 20, with summation over the test samples (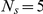), and given the model parameters 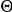 that minimized the mean square error between the model and the training data.

The paired difference between the test errors of both models was considered as an estimate of the performance of the extended model with position cost relative to the standard model with end cost only. Therefore, we performed a two-sided sign test under the null hypothesis that the paired differences ‘test error of standard model’ minus ‘test error of extended model’ have median zero, against the alternative that they do not have median zero at the 5% significance level.

## Results

### Responses in the one-target task

In the one-target task, subjects were asked to steer a virtual ball from the left side of the screen toward a single goal at the right (Figure 1a). As an example, figure 1 shows the ball position (panel c) and corresponding control (panel e) of a representative subject (S6) for 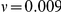. Each line represents a single trial (total 100 trials) starting at a position 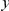 in the range between 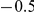 and 0.5.

**Figure 2:**
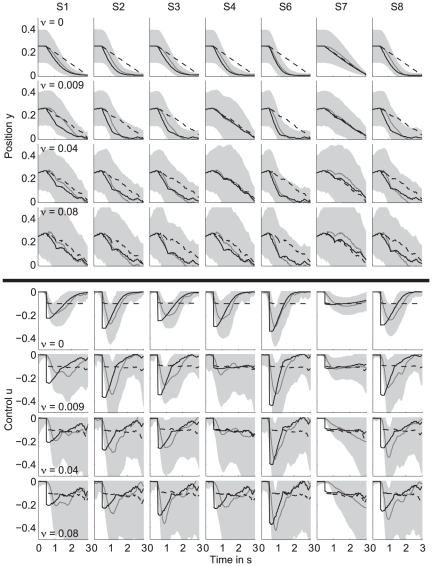
Behavior and model predictions for the one-target task. Top panels: average ball position displayed as mean (gray solid line) and standard deviation (gray shaded area) for all noise amplitudes (rows) and subjects (columns). The black dashed and solid line represent the average fit of the standard model and the extended model, respectively. Bottom panels: same for the control signal. Subject S5 was discarded (see Methods).

The average observed behavior was obtained by averaging the single trials for each subject and condition (Figure 2). Since the task was symmetric relative to 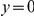, we did not find systematic differences in behavior between trials starting at 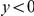 and 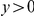 for the majority of subjects and conditions (see below). Therefore, we calculated the average over trials (solid gray line) by first inverting the sign of the position and corresponding control signal for trials starting at 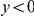 and then taking the average over all trials. The shaded area represents the standard deviation. Subject S4 showed different behavior between trials starting at 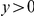 and trials starting at 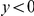 for 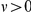. Therefore, the results of this subject should be interpreted as the average behavior between trials starting at 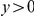 and trials starting at 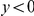.

Since subjects could only manipulate the vertical position of the ball, they always reached the horizontal target position (i.e., the right boundary of the screen) at 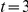 s. However, they were free in choosing the time at which they reached the vertical target position 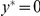. The time at which subjects reached the vertical target position differed substantially among subjects. This difference in behavior is most pronounced when considering the average ball trajectories for the noiseless condition (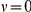, first row). Subject S6 is an example of a subject who reached 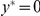 very early (at about 1.5 s) which was well before the final time of 3 s, resulting in an average ball trajectory that was curved. The corresponding control (fifth row) peaked just before 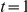 s and gradually decayed, reaching values close to zero after about 1.5 to 2 s. Subject S7 shows different behavior. This subject reached the target very close to the deadline 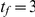 s by exerting a more or less constant control. The other subjects (S1–S4, S8) showed intermediate behavior, i.e., they reached 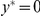 between 1.5 and 3 s.

The strategy subjects used to steer the ball toward the target is consistent across different noise levels for the majority of subjects (S1–S3, S6–S8). Subject S4 is an exception to this consistency since they reached the vertical target position relatively early in the noiseless condition, but reached the target close to the final time when the noise was increased (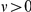). Note that all subjects had a reaction time of about 0.5 s before they started moving the ball.

### Model performance for the one-target task

The black dashed and solid line in Figure 2 represent the average model fit of the standard model and of the extended model with position cost, respectively. In the first 0.5 s, the control was set to zero to account for the subjects' reaction time. The majority of subjects reached the vertical target position well before the final time of 3 seconds. This behavior is inconsistent with the standard model (dashed line) which predicted a constant control and a straight trajectory reaching the target approximately at 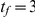 s. The predictions by the extended model (solid line) were in close agreement with the curved ball trajectory. This means that the position cost, which is a function of the distance between the ball and the target position during the trial, is essential to fit the behavior of the majority of the subjects.

Subject S7 (all noise levels) and subjects S4(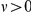) ended at the vertical target position close to the final time by applying a more or less constant control. This strategy was consistent with the standard model, where subjects minimized the control cost and end cost, but not the position cost. Fitting both models to the data of these subjects gave more or less the same result for both models. The reason is that for these cases the optimal value for parameter 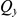 in the extended model (see equation 15) was close to zero, which reduced the extended model to the standard model.

**Figure 3:**
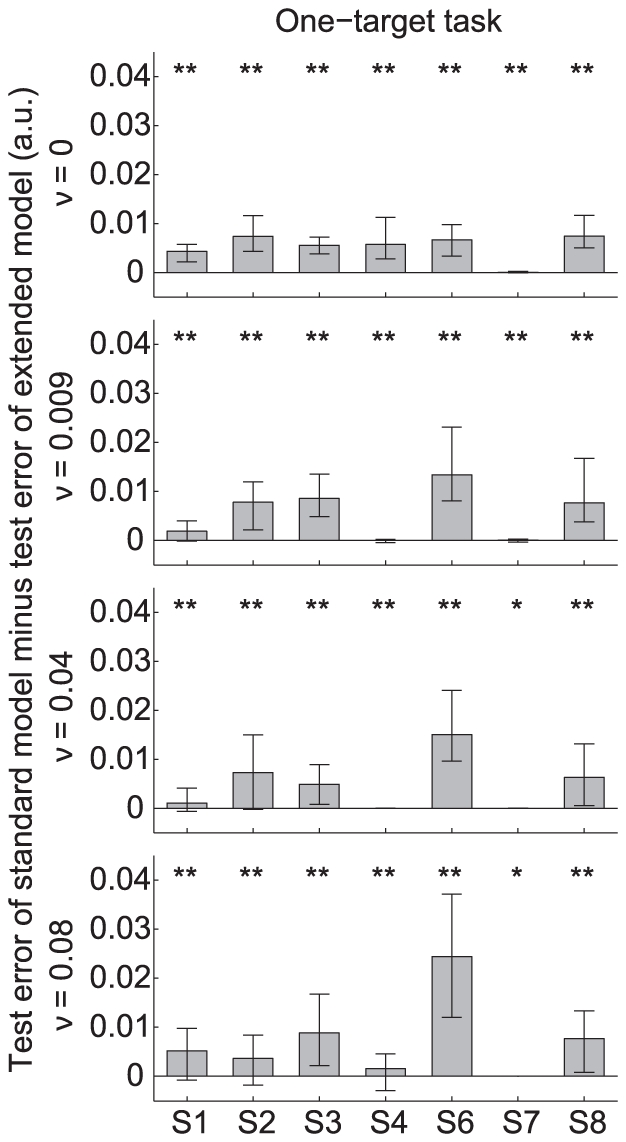
Model performance for the one-target task. Test error of standard model minus test error of extended model for all subjects and noise amplitudes. Values are given as the median over 100 cross-validation runs. The lower and upper error bars represent the 25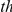 and 75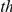 percentile, respectively. A positive value means that the extended model gave a better fit than the standard model. A value of zero means that there was no difference between the models. Conditions for which the extended model gave a significantly better prediction than the standard model are indicated by 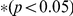 or 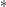 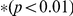. Subject S5 was discarded (see *Methods*).

To investigate whether the extended model with position cost gave a better prediction of the data than the standard model with end cost only, we computed the test error for each model which provided a measure how well the model fitted the data (see *Methods* for details). Figure 3 shows the test error of the standard model minus the test error of the extended model (‘test error difference’) for all subjects and noise amplitudes. Values are given as the median over 100 validation runs. The lower and upper error bars represent the 25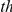 and 75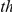 percentile, respectively. Thus, a positive value means that the extended model gave a better prediction than the standard model. A value of zero means that there was no difference between the models. Conditions for which the extended model gave a significantly better prediction than the standard model are indicated by 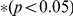 or 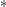 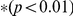.

For all subjects and noise amplitudes, the predictions by the extended model were better in agreement with the data than the predictions by the standard model, even when the test error difference was close to zero. The reason is that we used a non-parametric sign-test, which ranks the values according to their sign without making any assumptions on the underlying distribution. The results of Figure 3 reveal conditions for which the test error difference was positive and conditions for which this value was virtually zero. A positive test error difference means that including the position cost in the model improved the model prediction, corresponding to the strategy of steering the ball toward the desired target position well before the final time. A test error difference near zero means that the standard model with end cost only is sufficient to describe the observed behavior and that subjects steer the ball in a straight line reaching the target approximately at the time limit.

For subject S1–S3, S6 and S8 the test error difference was clearly positive for all noise amplitudes. Thus, for these subjects the behavior in the one-target task can best be described by a model which includes the position cost in its cost function, irrespective of the level of uncertainty in the task. Subject S4 showed a positive test error difference for 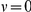, whereas for 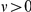 this value was virtually zero. Thus, this subject chose different strategies based on the level of uncertainty. For subject S7, the test error difference was virtually zero for all noise amplitudes, which means that including the position cost hardly improved the model prediction. This behavior was consistent across all noise amplitudes.

### Responses in the two-target task

In the previous sections, we found that a standard optimal control model with a control cost component and end cost component was insufficient to describe the behavior found in the one-target experiment for the majority of subjects. We found that the extended model gave a good fit of the data of the one-target experiment. Here, we investigate whether the extended model could also correctly predict the behavior in other time-constrained tasks. Therefore, we designed a second task in which subjects had to move the ball to either of two targets (see *Methods* for details). We selected a new group of seven subjects (S6–S12), three of which (S65–S8) also participated in the one-target task. The remaining four subjects (S9–S12) were naive with respect to the aim and procedure of the experiment to check whether the results of the second task could have been influenced by participation in the first task. As an example, Figure 1 shows the ball position (panel d) and corresponding control (panel f) of a representative subject (S6) for 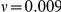. Each line represents a single trial (total 100 trials) starting at a position 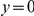.

**Figure 4:**
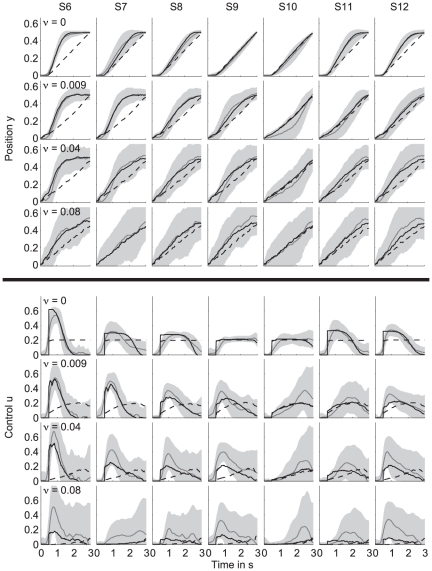
Behavior and model predictions for the two-target task. See figure 2 for details.

The average observed behavior was obtained by averaging the single trials for each subject and condition (Figure 4). Since the task was symmetric relative to 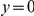, we did not find systematic differences in control for trials in which subjects aim for the upper and lower target, except for the sign. The only exception was subject S8 who steered the ball to the upper target only in the noiseless condition. We took the average over trials after inverting the sign of the position and corresponding control signals aiming for the lower target (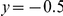), as if all trials were made to the upper target (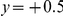). The gray line and shaded area represent the mean and standard deviation over 100 trials, respectively.

The time at which subjects reached the vertical target position differed substantially among subjects. This difference in behavior is most pronounced when considering the average ball trajectories for the noiseless condition (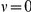, first row). Subject S6 is an example of a subject who reached 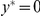 very early (at about 2 s) which was well before the final time of 3 s, resulting in an average ball trajectory that was curved. The corresponding control (fifth row) peaked at about 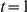 s and gradually decayed, reaching values close to zero after about 1.5 to 2 s. Subject S9 and S10 shows different behavior. These subjects reached the target very close to the deadline 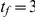 s, resulting in a straight path, by exerting a more or less constant control. The other subjects (S7, S8, S11, S12) showed intermediate behavior, i.e., they reached 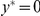 between 1.5 and 3 s.

The strategy subjects used to steer the ball toward the target is consistent across different noise levels for subjects S6, S8 and S12. Subjects S7 and S11 reached the vertical target position relatively early in the noiseless condition, but reached the target close to the final time when the noise was increased (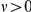). Subject S9 shows the opposite behavior. Subject S10 is consistent across different noise levels except for 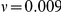 where this subject started moving the ball toward the target relatively late in the trial. All subjects had a reaction time of about 0.5 s before they started moving the ball.

### Model performance for the two-target task

The black dashed and solid line in Figure 4 represent the average model fit of the standard model and of the extended model with position cost, respectively. In the first 0.5 s, the control was set to zero to account for the subjects' reaction time. In this task, we found similar results as for the one-target task. The majority of subjects reached the vertical target position well before the final time of 3 seconds, which was inconsistent with the standard model (dashed line) but consistent with the extended model (solid line). This means that also for the two-target task, the position cost is essential to fit the behavior of the majority of the subjects. In some conditions subjects ended at the vertical target position close to the final time (e.g. S9, S10 for 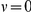; S7, S10 for 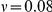), which was consistent with the standard model where subjects minimized the control cost and end cost, consistent with the standard model where subjects minimized the sum of control cost and end cost, and where no position cost appeared.

**Figure 5:**
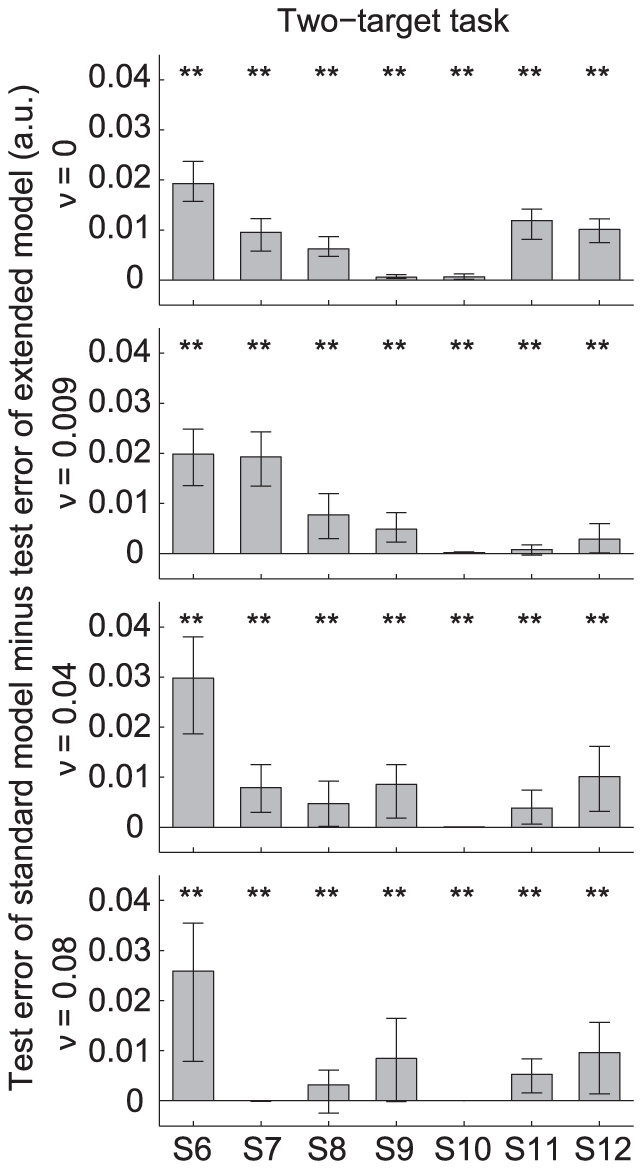
Model performance of the two-target task. See figure 3 for details.

To investigate whether the extended model gave a better prediction of the data than the standard model, we repeated the analysis that we used for the one-target task. Figure 5 shows the test error of the standard model minus the test error of the extended model for all subjects and noise amplitudes. Values are given as the median over 100 validation runs. The lower and upper error bars represent the 25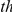 and 75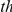 percentile, respectively. Conditions for which the extended model gave a significantly better prediction than the standard model are indicated by 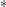 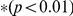.

For all subjects and noise amplitudes, the predictions by the extended model were significantly better in agreement with the data than the predictions by the standard model, even when the test error difference was close to zero. This result is in agreement with that of the one-target task (see Figure 3). For subjects S6, S8, S11 and S12 the test error difference was clearly positive for all noise amplitudes, corresponding to a strategy in which the ball was steered toward the desired target position well before 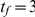 s. Subject S10 showed a test error difference close to zero for all noise amplitudes, corresponding to a strategy in which the ball moved in a straight line and arrived at the target position approximately at 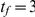 s. For subject S7, the test error difference was close to zero for 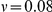, but positive for the other noise amplitudes. Considering the group of subjects which participated in both experiments (i.e., S6–S8), we conclude that for S6 and S8, the behavior in the two-target task was similar as for the one-target task.

## Discussion

In this study, we used optimal control theory to predict human motor behavior in two different time-constrained motor tasks. We investigated whether minimizing the usual cost function consisting of a control and end cost component is sufficient to describe the observed behavior. Therefore, we tested eight subjects in the one-target task in which they had to hit a target at a final time of 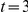 s. As a null hypothesis, we postulated the standard model which assumes that subjects minimize the integrated quadratic control and the squared distance between the ball and the target at the end of the trial. We extended the standard model by including a position cost component in the cost function, which penalized deviations between the position of the ball and the target throughout the trial. This cost component ensures that subjects steer the ball to the target position well before the final time. Both models were trained on a subset of the data to compute the model parameters, and tested on the remaining data to obtain the model performance. The results show that the majority of subjects steer the ball such that they reach the vertical target position well before the final time. This behavior was consistent across different noise levels. For all subjects and noise levels, the predictions by the extended model were better in agreement with data than the predictions by the standard model. This result rejects our hypothesis that a simple cost function representing a trade-off between effort and performance is sufficient to describe the observed behavior. To investigate whether the extended model is generic, we tested another group of subjects in the two-target task in which they had to hit one of two targets at the fixed final time. For this task, the predictions by the extended model were also in good agreement with the experimental data, much better than for the standard model.

Recently, Nagengast et al. [11] used a similar task in which subjects controlled a virtual ball undergoing Brownian motion (noise) towards a target that had to be reached at a final time of one second. Subjects were required to minimize an explicit cost that was a combination of the final positional error of the ball (end cost) and the integrated control cost. They proposed the use of a risk sensitive optimal controller that incorporated movement cost variance either as an added cost (risk-averse controller) or as an added value (risk-seeking controller). This raises the question whether our results could also be explained by risk sensitivity. Therefore, we fitted the risk sensitive model of Nagengast et al. to our data. For the conditions with stochastic dynamics (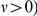, we reproduced their finding that this risk sensitive model fitted the data better than the risk-neutral model, which is equal to the standard model in this study (see *Appendix S1*). However, in our experiment, we also included a noiseless condition (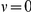) in which the movement of the ball was completely deterministic. For the noiseless condition, a risk sensitive model gives exactly the same predictions as a risk-neutral model. Our results show that for the noiseless condition, a risk-neutral model that minimizes the control and the final positional error is insufficient to predict the observed behavior. Thus, our results cannot be explained by risk sensitivity.

How can we explain that subjects show a different behavior in the task of Nagengast et al. and our one-target task, while both tasks were rather similar? In the experiment of Nagengast et al., subjects received feedback about the control cost and end cost during each trial, and about the average total cost across trials. Their subjects were requested to minimize the sum of the control cost and end cost, corresponding to the standard model. If subjects would steer the ball to the vertical target position before the final time, their control cost would increase. Since subjects were requested to minimize the control cost and end cost, they adjusted their behavior such that it was in agreement with the standard model. In addition, although their task was very similar to ours, the dynamics of the system differed substantially. We used a first order dynamics to relate the control to the ball position, that is, a control action changed the vertical velocity of the ball (equation 1). Nagengast et al. [11] used a second order dynamics in which a control action acted as a force on a frictionless mass (the ball) causing it to accelerate or decelerate. In our experiment, subjects could move the ball with the joystick toward a desired position, which could then be maintained by simply exerting no control. In their experiment, subjects could move the ball toward a desired position, but in order to maintain that position, subjects had to decelerate the ball by exerting control opposite to the movement direction. This may have affected the subjects' control strategy.

Braun et al. [23] have used a cumulative position-dependent cost function to model reaching tasks. In their study no final time was assumed, but instead, the movement time resulted as a consequence of the cumulative position cost. Even though they used an infinite horizon model, it suggests that the cumulative position cost may have the same effect as in our study. Therefore, we implemented an infinite horizon model in our experiments (see *Appendix S1*). We found that the optimal control in the infinite horizon model is similar to the optimal control in the finite horizon model. This demonstrates that introducing a position cost consistently predicts that subjects steer the ball such that it arrives at the vertical target position before the final time, irrespective of the time horizon.

In our experiment, we asked subjects to hit the target but we did not gave any instructions as to whether the movement should meet any criteria. This raises the question why subjects would minimize the position error over time. Todorov [2] stated that the cost that is relevant to the sensorimotor system may not directly correspond to our intuitive understanding of ‘the task’ and so its detailed form should be considered a relatively free parameter. Thus, the explicit instruction to the subjects of the task's goal may not necessarily reflect the goal that the sensorimotor system implicitly tries to achieve. This is consistent with our results showing that the subjects' strategy can be predicted by a model that also minimizes the position cost, although this was not an explicit aim of the task.

However, this does not explain what the cause of this position cost might be. One likely explanation is that maintaining a constant control throughout the trial is more difficult than producing a large control in the beginning of the trial and some corrective movements at the end. Thus, if the trial duration is large, a control strategy which spreads the control equally over the available time would probably be extremely difficult to achieve, even though that this strategy is the optimal strategy according to the standard model. The trial duration in our experiments was set to 3 s, which is rather long for a typical hand or arm movement. This suggests that a relatively long trial duration cause subjects to move faster toward the target compared to short trial durations. This hypothesis could be tested in future work. An alternative explanation is that subjects avoid the risk of missing the target and therefore steer the ball toward the target before the final time.

One can argue that including a third component in the cost function of the extended model will by itself give a better prediction since it adds two additional free parameters compared to the standard model (i.e., 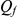 for the standard model; 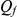, 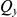 and 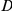 for the extended model; 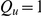 for both models). However, the model predictions do not depend on the number of free parameters since we applied cross-validation to both models. The additional free parameters in the extended model could cause so-called overfitting when these parameters were not relevant for the task. In that case, the extended model would give a worse fit than the standard model, resulting in negative values of the test error differences in figures 3 and 5. At the contrary, these figures show that on average, the extended model gives a significantly better prediction than the standard model.

The extended model includes one fixed parameter, the sensorimotor delay 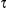, which was set to 200 ms (see *Methods*). To investigate the sensitivity of the model to this parameter, we also did the cross-validation procedure for 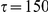 ms, which was equal to the value chosen by Nagengast et al. [11]. Similar to the extended model with 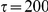 ms, we calculated the test error of the standard model minus the test error of the extended model, yielding 100 error values for each of 64 conditions (8 subjects
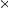
4 noise amplitudes
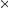
2 tasks). For each condition, we tested whether the median obtained by the model with 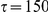 ms was equal to the median obtained by the model with 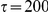 ms (Mann-Whitney U-test). For six conditions, we found a significant difference (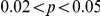). For the remaining 58 conditions, the difference was not significant (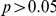). These results show that the extended model is not sensitive to the precise value of the sensorimotor delay within the range of 150 to 200 ms for the majority of conditions tested in this study.

In our model, we included a quadratic end cost and a position cost which was quadratic only locally (around the target position) and leveled off further away from the target to reduce the effect of outliers. Note that in this experiment, the difference in model prediction between a quadratic or a locally quadratic end cost function will be small, since the ball's final position is scattered around the target position (see figures 2 and 4, first row) which is within the quadratic region of both functions. The position cost, however, depends on the ball position relative to the target position throughout the trial. Initially, the ball's position is in general relatively far from the desired target position, resulting in large errors. For sensorimotor learning, it has been shown that people use a cost function which increases approximately quadratically for small errors but less than quadratically for large errors [19]. Therefore, we used a tanh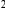-function (equation 13 and 14) which fulfills these criteria and which sets an upper bound on the position cost.

For the two-target task, the standard model gives an additional, more subtle theoretical prediction. In the beginning of the trial, it is best to steer towards 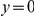 (between the targets) and delay the choice which target to aim for if the noise amplitude is large. In our experiments, the standard model predicts only a very small control toward 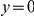 in the first 0.5 seconds, even for the highest noise amplitude (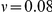). Thus, according to the standard model this so-called symmetry breaking is barely measurable in our experiments. Moreover, the symmetry breaking vanishes when the position cost is introduced. This explains why we found that subjects never steered the ball away from the target towards 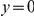 during the first part of the trial, even not for the highest noise amplitude.

The results of our study show that a model that only minimizes control effort and maximizes performance cannot describe human motor behavior in a time-constrained sensorimotor task. A model that also accounts for the deviation from the goal throughout the task execution gives a significantly better fit the observed behavior.

## Supporting Information

Appendix S1 **Risk sensitive and infinite horizon models.** In this appendix, we derive a risk sensitive optimal control model for the one-target and two-target task. We discuss the model performance. As an alternative to the finite horizon models presented in this study, we discuss the application of infinite horizon models.
(PDF)
