# Supplementary material for: Time-Integrated Position Error Accounts for Sensorimotor Behavior in Time-Constrained Tasks
Source: PLoS One. 2012 Mar 21;7(3):e33724. doi: 10.1371/journal.pone.0033724 (PMC3312345; doi:10.1371/journal.pone.0033724)
Supplement: Appendix S1 — Risk sensitive and infinite horizon models. In this appendix, we derive a risk sensitive optimal control model for the one-target and two-target task. We discuss the model performance. As an alternative to the finite horizon models presented in this study, we discuss the application of infinite horizon models. (PDF) [file pone.0033724.s001.pdf]

Appendix S1

A. Derivation of the risk sensitive model

In this section, we derive the risk sensitive optimal control for the one-target and two-target task. For the basic model with end cost only, this solution can be obtained analytically. The dynamics of the control problem is given by the stochastic differential equation $dy = u(y,t)dt + d\xi$ (see equation 1). The total cost function of this control problem is equal to the sum of an end cost function and a cumulative control cost function, resulting in

$$C_{total} = \left\langle C_f(y_f) + \int_0^{t_f} \tfrac{1}{2} Q_u u(y(t),t)^2 dt \right\rangle_{y_0},$$

(see equation 4) with end cost function

$$\begin{aligned} C_f(y_f) &= \begin{cases} \frac{1}{2}Q_f(y_f+a)^2 & \text{if } y_f \le 0 \\ \frac{1}{2}Q_f(y_f-a)^2 & \text{if } y_f > 0 \end{cases} \\ &= \tfrac{1}{2}Q_f(|y_f|-a)^2 \end{aligned}$$

with $a \ge 0$. Note that $a = 0$ gives the control problem for the one-target task and $a = 0.5$ for the two-target task. As derived in [1], the optimal cost-to-go satisfies

$$J_\theta(y,t) = \begin{cases} \langle C_f(y_f)\rangle_y & \text{if } \theta = \frac{1}{\nu Q_u} \\ -\frac{\nu Q_u}{1-\theta\nu Q_u} \ln Z_\theta(y,t) & \text{if } \theta \ne \frac{1}{\nu Q_u} \end{cases}$$

where

$$Z_\theta(y,t) = \left\langle \exp\left(-\frac{1-\theta\nu Q_u}{\nu Q_u} C_f(y_f) \right) \right\rangle_y$$

and y represents the path for uncontrolled dynamics. A general derivation of the optimal cost-to-go in stochastic optimal control problems can be found in [1]. First, we consider the case $\theta = \frac{1}{\nu Q_u}$. For this case, the optimal cost-to-go can be rewritten by substituting the end cost function, which yields

$$\begin{aligned} J_\theta(y,t) &= \left\langle \tfrac{1}{2}Q_f\big(|y_f|-a\big)^2 \right\rangle_y \\ &= \tfrac{1}{2}Q_f\Big(\langle y_f^2\rangle_y - 2a\langle |y_f|\rangle_y + a^2\Big). \end{aligned}$$

The expectation value $\langle y_f^2\rangle_y$ is given by

$$\langle y_f^2\rangle_y = \left\langle \Big(y + \int_t^{t_f} d\xi\Big)^2 \right\rangle_y = y^2 + \nu(t_f - t).$$

The distribution of $|y_f|$ conditioned on y is a reflected Brownian motion [2] and reads

$$\mathbb{P}\big(|y_f| \in dy' \big| y\big) = \frac{1}{\sqrt{2\pi\nu(t_f - t)}}\Bigg(\exp\bigg(-\frac{(|y| + y')^2}{2\nu(t_f - t)}\bigg) + \exp\bigg(-\frac{(|y| - y')^2}{2\nu(t_f - t)}\bigg)\Bigg) dy' \qquad (y \geq 0).$$

We use this distribution to find the expectation value $\langle |y_f| \rangle_y$, which is given by

$$\begin{aligned}\langle |y_f| \rangle_y &= \int_0^\infty y' \mathbb{P}\big(|y_f| \in dy' \big| y\big) \\ &= \frac{2\nu(t_f - t)}{\sqrt{2\pi\nu(t_f - t)}} \exp\bigg(-\frac{y^2}{2\nu(t_f - t)}\bigg) + y \ \mathrm{erf}\bigg(\frac{y}{\sqrt{2\nu(t_f - t)}}\bigg).\end{aligned}$$

The optimal control u^* is proportional to the partial derivative of the optimal cost-to-go $J_\theta(y, t)$ to y and is given by

$$\begin{aligned}u^*(y, t) &= -\frac{1}{Q_u}\frac{\partial}{\partial y} J_\theta(y, t) \\ &= \frac{Q_f}{Q_u}\bigg(\mathrm{erf}\bigg(\frac{y}{\sqrt{2\nu(t_f - t)}}\bigg) a - y\bigg).\end{aligned}$$

Next, we consider the case $\theta \neq \frac{1}{\nu Q_u}$. $Z_\theta(y, t)$ is a path integral that satisfies

$$\begin{aligned}Z_\theta(y, t) &= \bigg\langle \exp\bigg(-\frac{Q_f(1 - \theta\nu Q_u)}{2\nu Q_u}(|y_f| - a)^2\bigg)\bigg\rangle_y \\ &= \int_0^\infty \exp\bigg(-\frac{Q_f(1 - \theta\nu Q_u)}{2\nu Q_u}(y' - a)^2\bigg)\mathbb{P}\big(|y_f| \in dy' \big| y\big) \\ &= \psi_-(y, t) + \psi_+(y, t)\end{aligned}$$

where

$$\psi_\pm(y, t) = \frac{1}{\sqrt{2\pi\nu(t_f - t)}}\int_0^\infty \exp\bigg(-\frac{Q_f(1 - \theta\nu Q_u)}{2\nu Q_u}(y' - a)^2 - \frac{(|y| \pm y')^2}{2\nu(t_f - t)}\bigg) dy'.$$

We define two new functions:

$$K(t) = \frac{Q_f}{Q_u + (1 - \theta\nu Q_u)Q_f(t_f - t)}$$

and

$$\mu_\mp(y, t) = \frac{K(t)Q_u}{Q_f}\Big(\mp |y| + (1 - \theta\nu Q_u)Q_f Q_u^{-1}(t_f - t)a\Big).$$

We use these functions to rewrite the expression for $\psi_\pm(y,t)$, which yields

$$\begin{aligned}\psi_\pm(y,t) &= \frac{1}{\sqrt{2\pi\nu(t_f-t)}}\exp\bigg(-\frac{K(t)(1-\theta\nu Q_u)}{2\nu}(|y|\pm a)^2\bigg)\int_0^\infty \exp\bigg(-\frac{Q_f(y'-\mu_\mp(y,t))^2}{2\nu(t_f-t)K(t)Q_u}\bigg)dy'\\ &= \sqrt{\frac{K(t)Q_u}{Q_f}}\exp\bigg(-\frac{K(t)(1-\theta\nu Q_u)}{2\nu}(|y|\pm a)^2\bigg)\bigg(\tfrac{1}{2}+\tfrac{1}{2}\mathrm{erf}\bigg(\sqrt{\frac{Q_f}{2\nu(t_f-t)K(t)Q_u}}\mu_\mp(y,t)\bigg)\bigg)\end{aligned}$$

under the condition that

$$0 < Q_u + (1-\theta\nu Q_u)Q_f(t_f-t).$$

Otherwise $\psi_\pm(y,t)=\infty$. The optimal control is given by

$$\begin{aligned}u^*(y,t) &= \frac{\partial}{\partial y}\frac{\nu}{1-\theta\nu Q_u}\ln Z_\theta(y,t)\\ &= Z_\theta(y,t)^{-1}\bigg(\frac{\nu}{1-\theta\nu Q_u}\frac{\partial}{\partial y}\psi_-(y,t)+\frac{\nu}{1-\theta\nu Q_u}\frac{\partial}{\partial y}\psi_+(y,t)\bigg).\end{aligned}$$

Finally, we rewrite the optimal control in terms of $K(t)$ and $\psi_\pm(y,t)$. Let

$$\begin{aligned}&\frac{\nu}{1-\theta\nu Q_u}\frac{\partial}{\partial y}\psi_\pm(y,t)\\ &\quad= -K(t)\big(y\pm a\ \mathrm{sign}(y)\big)\psi_\pm(y,t)\mp\\ &\quad\frac{\mathrm{sign}(y)}{\sqrt{2\pi\nu(t_f-t)}}\frac{K(t)Q_u}{Q_f}\frac{\nu}{1-\theta\nu Q_u}\exp\bigg(-\frac{K(t)(1-\theta\nu Q_u)(|y|\pm a)^2}{2\nu}-\frac{Q_f\mu_\mp(y,t)^2}{2\nu(t_f-t)K(t)Q_u}\bigg)\\ &\quad= -K(t)\big(y\pm a\ \mathrm{sign}(y)\big)\psi_\pm(y,t)\mp\\ &\quad\frac{\mathrm{sign}(y)}{\sqrt{2\pi\nu(t_f-t)}}\frac{K(t)Q_u}{Q_f}\frac{\nu}{1-\theta\nu Q_u}\exp\bigg(-\frac{|y|^2}{2\nu(t_f-t)}-\frac{1-\theta\nu Q_u}{2\nu Q_u}Q_f a^2\bigg).\end{aligned}$$

After substituting this equation we find

$$u^*(y,t) = -K(t)\bigg(y+a\ \mathrm{sign}(y)\frac{-\psi_-(y,t)+\psi_+(y,t)}{\psi_-(y,t)+\psi_+(y,t)}\bigg).$$

We can verify that this equation equals the case $\theta=\frac{1}{\nu Q_u}$, since

$$\lim_{\theta\to\frac{1}{\nu Q_u}}\psi_\pm(y,t) = \tfrac{1}{2}+\tfrac{1}{2}\mathrm{erf}\bigg(\frac{\mp|y|}{\sqrt{2\nu(t_f-t)}}\bigg).$$

The optimal control for the case $\theta=\frac{1}{\nu Q_u}$ is given by

$$\lim_{\theta \to \frac{1}{\nu Q_u}} u^*(y,t) = -\frac{Q_f}{Q_u}\left(y - a\ \mathrm{sign}(y)\ \mathrm{erf}\left(\frac{|y|}{\sqrt{2\nu(t_f - t)}}\right)\right)$$
$$= -\frac{Q_f}{Q_u}\left(y - a\ \mathrm{erf}\left(\frac{y}{\sqrt{2\nu(t_f - t)}}\right)\right).$$

Note that the optimal control for the standard model is found by taking $\theta = 0$.

B. Model performance of the risk sensitive model

In the standard model, the performance criterion of minimizing the expected cost-to-go (equation 4 of the main article) assumes that the certainty equivalent, i.e., the maximal cost one is willing to pay for certain rather than the uncertain cost associated to the control problem, equals the expected cost-to-go. The resulting control problem is said to be risk neutral. When the certainty equivalent is higher or lower than the expected cost-to-go, the performance criterion is adjusted to minimize an exponentially weighted cost-to-go [3]:

$$\frac{1}{\theta} \ln \left\langle \exp\left(\theta C_f(y_f) + \theta \int_0^{t_f} \tfrac{1}{2} Q_u u(y(t'), t')^2 dt'\right)\right\rangle_{y_0} \tag{S1}$$

where θ is a parameter that quantifies the risk sensitivity. If θ is negative then the certainty equivalent is lower than the expected cost-to-go and the controller is said to be risk seeking, and if θ is positive then the certainty equivalent is higher than the expected cost-to-go and the controller is said to be risk averse, and the case $\theta = 0$ is the risk neutral case [4].

In the control task with one target located at zero, we choose an end cost function that is quadratic around the target location (equation 3 with $y^* = 0$). The optimal control is given by

$$u^*(y,t) = -K(t)y$$

with

$$K(t) = \frac{Q_f}{Q_u + (1 - \theta\nu Q_u)Q_f(t_f - t)} \tag{S2}$$

under the condition that

$$0 < Q_u + (1 - \theta\nu Q_u)Q_f(t_f - t), \tag{S3}$$

otherwise no optimal control exists (see section A). In the control task with two targets located at -0.5 and $+0.5$, we choose an end cost function that is quadratic around the target locations (equation 8). The optimal control is given by

$$u^*(y,t) = -K(t)\big(y - \overline{a}\big) \tag{S4}$$

where $K(t)$ is as given by equation S2,

$$\overline{a} = -a \ \mathrm{sign}(y)\frac{-\psi_-(y,t) + \psi_+(y,t)}{\psi_-(y,t) + \psi_+(y,t)},$$

with $a = 0.5$ and

$$\begin{aligned}\psi_\pm(y,t) &= \sqrt{\tfrac{K(t)Q_u}{Q_f}} \exp\Big(- \tfrac{K(t)(1-\theta\nu Q_u)}{2\nu}(|y| \pm a)^2\Big)\Big(\tfrac{1}{2} + \tfrac{1}{2}\mathrm{erf}\Big(\sqrt{\tfrac{Q_f}{2\nu(t_f-t)K(t)Q_u}}\mu_\mp(y,t)\Big)\Big)\\ &= \tfrac{K(t)Q_u}{Q_f}\Big(\mp |y| + (1-\theta\nu Q_u)Q_f Q_u^{-1}(t_f - t)a\Big),\end{aligned}$$

and under the condition (S3) for the optimal control to exist (see section A for a derivation). We obtain the optimal control for the case that $\theta = \frac{1}{\nu Q_u}$ by taking the limit:

$$\lim_{\theta\to 1/\nu Q_u} u^*(y,t) = -\frac{Q_f}{Q_u}\left(y - a \ \mathrm{erf}\left(\frac{y}{\sqrt{2\nu(t_f - t)}}\right)\right).$$

We consider the risk sensitive model as an alternative to the extended model to explain the subjects' behavior. For the noiseless condition ($\nu = 0$) the dynamics is completely deterministic and the risk sensitive model reduces to the standard model: in the absence of noise, the expectation value in equation S1 vanishes and the exponentially weighted cost reduces to the total cost in the standard model. Therefore, we do not include the noiseless condition in this section. Note that the extended model does give a different prediction than the standard model for $\nu = 0$. The results show that the extended model yields a significantly better prediction than the standard model (figures 3 and 5 of the main article).

For a noise amplitude $\nu > 0$, the risk sensitive model gives different predictions than the standard model. Figure S1 shows the model performance of the risk sensitive model compared to the standard model for the one-target (left panel) and two-target task (right panel). Values are given as the median over 100 cross-validation runs. The lower and upper error bars represent the 25th and 75th percentile, respectively. Conditions for which the test error of the standard model was significantly different from the test error of the risk sensitive model (two-sided sign test, $\alpha = 0.05$) are indicated by $**$ ($p < 0.01$). For all subjects and tasks, the test error of the standard model minus the test error of the risk sensitive model is significantly larger than zero. Thus, the risk sensitive model gives a significantly better prediction of the subjects' behavior than the standard model.

Figure S2 shows the value of the risk-sensitivity parameter θ for all subjects in the one-target (top panel) and two-target task (bottom panel). Values are given as the median over 100 cross-validation runs. The lower and upper error bars represent the 25th and 75th percentile, respectively. Subsequent data points in triplets correspond to noise amplitudes ν of 0.009, 0.04 and 0.08, respectively. For the majority of the subjects, the risk sensitivity decreases from highly risk sensitive for $\nu = 0.009$ to approximately risk neutral for $\nu = 0.08$. From these observations we conclude that the risk sensitive model can describe

the subject behavior at nonzero noise levels, although it would require a risk sensitivity that depends on the noise level.

C. Infinite horizon models

The stochastic optimal control model considered by Braun et al. [5] is discrete in time, has an infinite time horizon, a dynamics of the form

$$y_{t+1} = y_t + u_t + \text{“signal-dependent noise”},$$

and a cost function of the form

$$\text{Cost } J = \tfrac{1}{2}\left\langle \sum_{t=0}^{\infty} \left(Qy_t^2 + Ru_t^2\right) \right\rangle, \tag{S5}$$

where Q and R are constants. The noise is signal-dependent [6], which means that the noise is zero when the state y is zero. This implies that if the state is zero ($y_t = 0$) then it is optimal to perform zero control ($u_t = 0$) at the present time (t) and all future times, because by doing so the state will remain zero ($y_{t+1} = y_t + u_t + \text{“noise”} = 0 + 0 + 0$), and the contribution to the cost is zero ($Qy_t^2 + Ru_t^2 = 0$) when the state and the control are zero. An important consequence of using signal-dependent noise is that the cost will not blow up as time proceeds.

If the noise is not signal-dependent, as it is in our experiments, the cost (equation S5) will blow up either due to $\sum_{t=0}^{\infty}\langle Qy_t^2\rangle$ blowing up because the state is perturbed by noise that is not sufficiently corrected for by the control, or due to $\sum_{t=0}^{\infty}\langle Ru_t^2\rangle$ blowing up because perturbations of the state due to noise are too much corrected for. Therefore, we consider an alternative infinite horizon model without signal-dependent noise. The dynamics of the control problem are described by

$$dy_t = u(t)dt + d\xi$$

with a cost function

$$C_{total} = \lim_{t_f \to \infty} \frac{1}{t_f} \left\langle \int_0^{t_f} \tfrac{1}{2} Q_u u(t)^2 dt + \int_0^{t_f} \tfrac{1}{2} Q_y V(y(t)) dt \right\rangle_{y_0}.$$

This is the expected cost in the finite horizon model with end time t_f, but with no end cost and averaged over time and in the limit where the end time goes to infinity. The cost function optimized over the control is the optimal expected cost-to-go J and satisfies the Hamilton-Jacobi-Bellman equation

$$\min_u \left(\tfrac{1}{2} Q_u u^2 + \tfrac{1}{2} Q_y V + u \frac{\partial}{\partial y} J + \tfrac{1}{2} \nu \frac{\partial^2}{\partial y^2} J \right) = c$$

where c is some constant [7]. It follows that the optimal control u^* satisfies

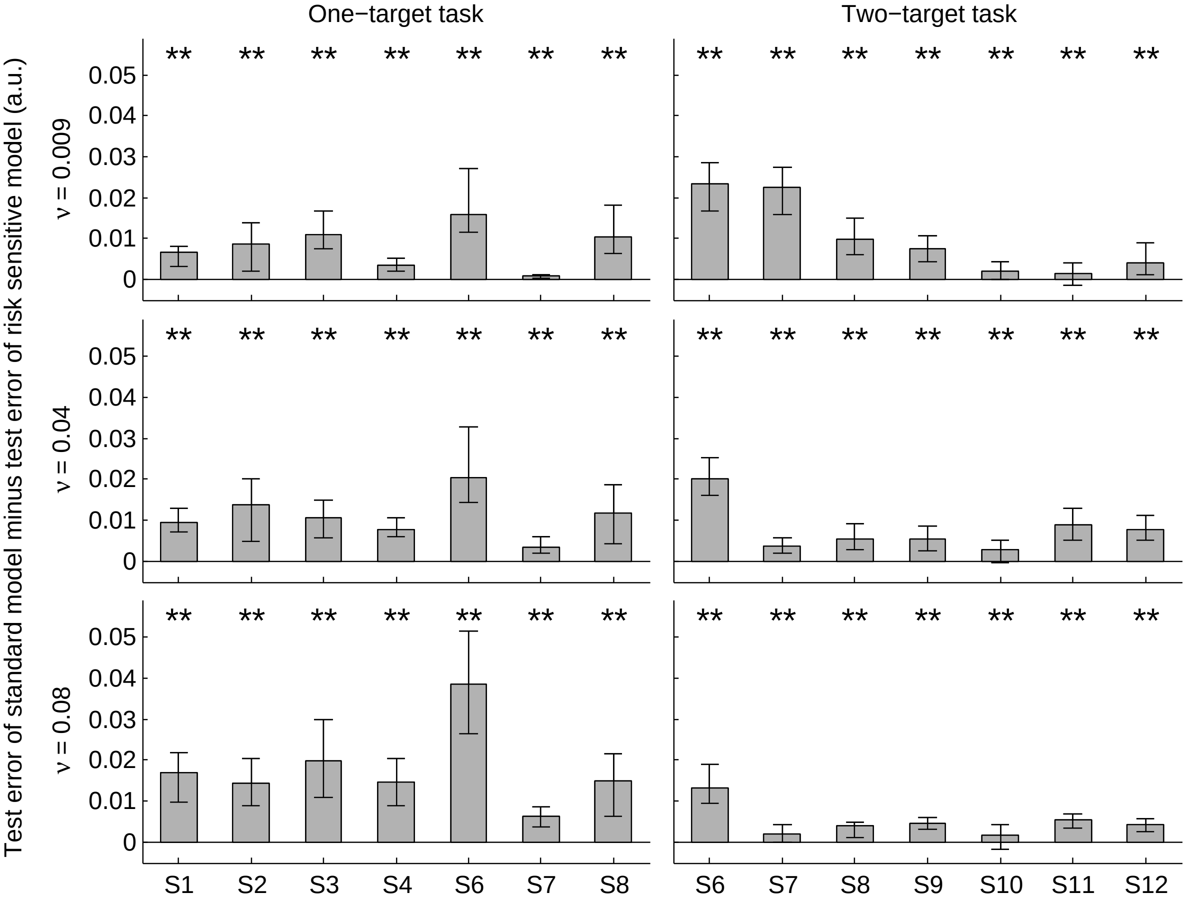

Figure S1. Model performance of the risk sensitive model. Test error of standard model minus test error of risk sensitive model ('test error difference') for all subjects and noise amplitudes, for the one-target (left panel) and two-target task (right panel). Subject S5 has been discarded. Values are given as the median over 100 cross-validation runs. The lower and upper error bars represent the 25th and 75th percentile, respectively. Conditions for which the test error of the standard model was significantly different from the test error of the risk sensitive model (two-sided sign test, $\alpha = 0.05$) are indicated by $**$ ($p < 0.01$).

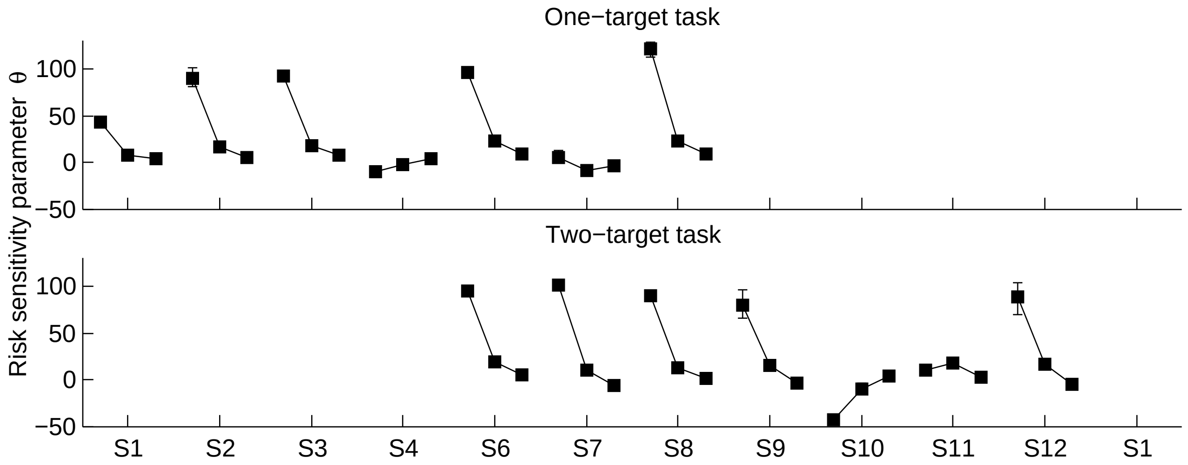

Figure S2. Risk sensitivity. Value of the risk-sensitivity parameter θ in the one-target (top panel) and two-target task (bottom panel). Subject S5 has been discarded. Values are given as the median over 100 cross-validation runs. The lower and upper error bars represent the 25th and 75th percentile, respectively. Each subsequent data point corresponds to a noise amplitude ν of 0.009, 0.04 and 0.08, respectively.

$$u^* = -Q_u^{-1} \frac{\partial}{\partial y} J \tag{S6}$$

and that the optimal expected cost-to-go J satisfies the HJB equation

$$-\tfrac{1}{2} Q_u^{-1} \Big(\frac{\partial}{\partial y} J \Big)^2 + \tfrac{1}{2} Q_y V + \tfrac{1}{2} \nu \frac{\partial^2}{\partial y^2} J = c.$$

Since the path cost function V does not depend on time, there is no explicit time dependence in the HJB equation, hence its solution J will also not explicitly depend on time. In the one-target task, we choose a path cost of the form

$$V(y) = \big(\tanh Dy \big)^2,$$

where D is a constant. Using the relations

$$\begin{aligned} \frac{\partial}{\partial y} \ln \cosh Dy &= D \tanh Dy \\ \frac{\partial^2}{\partial y^2} \ln \cosh Dy &= D^2 \frac{1}{(\cosh Dy)^2} \\ &= D^2 \big(1 - (\tanh Dy)^2 \big), \end{aligned}$$

one verifies by substitution that the optimal expected cost-to-go is given by

$$J(y) = G \log \cosh Dy$$

with

$$G = -\tfrac{1}{2} Q_u \nu D + \tfrac{1}{2} \sqrt{Q_u^2 \nu^2 D^2 + 4 D^{-1} Q_u Q_y}. \tag{S7}$$

Note that G is positive definite, unless the path cost parameter Q_y equals zero, then G also equals zero and the optimal control is to perform no action. The optimal control follows from equation S6 and is given by

$$u^*(y) = -Q_u^{-1} G D \tanh Dy.$$

In the two-target task, we choose a path cost function of the form

$$V(y) = \begin{cases} \Big(\tanh\big(D(y+a)\big)\Big)^2 & \text{if } y \leq 0 \\ \Big(\tanh\big(D(y-a)\big)\Big)^2 & \text{if } y > 0 \end{cases}$$

where the targets are located at $y = -a$ and $y = a$. One verifies in a similar way as in the one-target case that the optimal expected cost-to-go is given by

$$J(y) = \begin{cases} G \log \cosh D(y+a) & \text{if } y \leq 0 \\ G \log \cosh D(y-a) & \text{if } y > 0 \end{cases}$$

with G given by equation S7. The optimal control follows from equation S6 and is given by

$$u^*(y) = \begin{cases} -Q_u^{-1} G D \tanh D(y+a) & \text{if } y \leq 0 \\ -Q_u^{-1} G D \tanh D(y-a) & \text{if } y > 0. \end{cases}$$

Note that the optimal control shows no symmetry breaking: it is always optimal to steer towards the nearest target. The optimal control in the infinite-horizon model with path cost is similar to the optimal control in the finite horizon model with path cost. When the noise level ν is low, in either model the pace in which to move towards a target is dominated by the path cost, which means for both models the optimal behavior is to arrive at the target before the end time. When the noise level is high, then in either model we find that the control in absolute value is fairly small, which is explained by the fact that the influence of the noise is strong relative to the control.

References

1. Kappen HJ (2005) Path integrals and symmetry breaking for optimal control theory. J Stat Mech-Theory E 2005: P11011.

2. Karatzas I, Shreve SE (2000) Brownian motion and stochastic calculus. Springer.

3. Fleming WH (2006) Risk sensitive stochastic control and differential games. Commun Inf Syst 6: 161–178.

4. van den Broek B, Wiegerinck W, Kappen HJ (2010) Risk sensitive path integral control. Proc UAI-10 : 615–622.

5. Braun DA, Ortega PA, Wolpert DM, Friston KJ (2009) Nash equilibria in multi-agent motor interactions. PLoS Comput Biol 5: e1000468.

6. Harris CM, Wolpert DM (1998) Signal-dependent noise determines motor planning. Nature 394: 780–784.

7. Borkar VS (2005) Controlled diffusion processes. Probability Surveys 2: 213–244.
